# Metabolic Roles of Androgen Receptor and Tip60 in Androgen-Dependent Prostate Cancer

**DOI:** 10.3390/ijms21186622

**Published:** 2020-09-10

**Authors:** Kah Ni Tan, Vicky M. Avery, Catalina Carrasco-Pozo

**Affiliations:** 1 Discovery Biology, Griffith Institute for Drug Discovery, Griffith University, Nathan, QLD 4111, Australia; kahni.tan@griffith.edu.au (K.N.T.); v.avery@griffith.edu.au (V.M.A.); 2 CRC for Cancer Therapeutics, Griffith Institute for Drug Discovery, Griffith University, Nathan, QLD 4111, Australia

**Keywords:** androgen receptor, Tip60, prostate cancer, metabolism, c-Myc, HIF-1α, p53

## Abstract

Androgen receptor (AR)-mediated signaling is essential for the growth and differentiation of the normal prostate and is the primary target for androgen deprivation therapy in prostate cancer. Tat interactive protein 60 kDa (Tip60) is a histone acetyltransferase that is critical for AR activation. It is well known that cancer cells rewire their metabolic pathways in order to sustain aberrant proliferation. Growing evidence demonstrates that the AR and Tip60 modulate key metabolic processes to promote the survival of prostate cancer cells, in addition to their classical roles. AR activation enhances glucose metabolism, including glycolysis, tricarboxylic acid cycle and oxidative phosphorylation, as well as lipid metabolism in prostate cancer. The AR also interacts with other metabolic regulators, including calcium/calmodulin-dependent kinase kinase 2 and mammalian target of rapamycin. Several studies have revealed the roles of Tip60 in determining cell fate indirectly by modulating metabolic regulators, such as c-Myc, hypoxia inducible factor 1α (HIF-1α) and p53 in various cancer types. Furthermore, Tip60 has been shown to regulate the activity of key enzymes in gluconeogenesis and glycolysis directly through acetylation. Overall, both the AR and Tip60 are master metabolic regulators that mediate cellular energy metabolism in prostate cancer, providing a framework for the development of novel therapeutic targets in androgen-dependent prostate cancer.

## 1. Introduction

Prostate cancer is the second most common cancer among men, with an estimated 1.3 million new cases worldwide in 2018 [1]. Prostate-specific antigen (PSA) is routinely used as a biomarker for the diagnosis, prognosis, and monitoring of disease progression in prostate cancer, although the benefit of PSA testing remains controversial [2]. A rapid increase in serum PSA has been linked to early disease progression and to a decreased overall survival, whereas a persistent undetectable PSA level after radical prostatectomy is associated with prolonged disease-free survival [3]. Although the commercial availability of PSA testing allows for early detection and treatment, current treatment options can only palliate symptoms and extend patients’ life expectancy. Androgen deprivation therapy is typically used as the first-line treatment in patients with advanced or metastatic prostate cancer [4]. Even though androgen deprivation therapy provides remission during the early stage of treatment, most patients eventually experience recurrence despite castrate levels of serum androgen [5]. This incurable stage of prostate cancer is known as castrate-resistant prostate cancer and is associated with poor survival of only 9–13 months in those who develop metastases [6].

**Figure 1:**
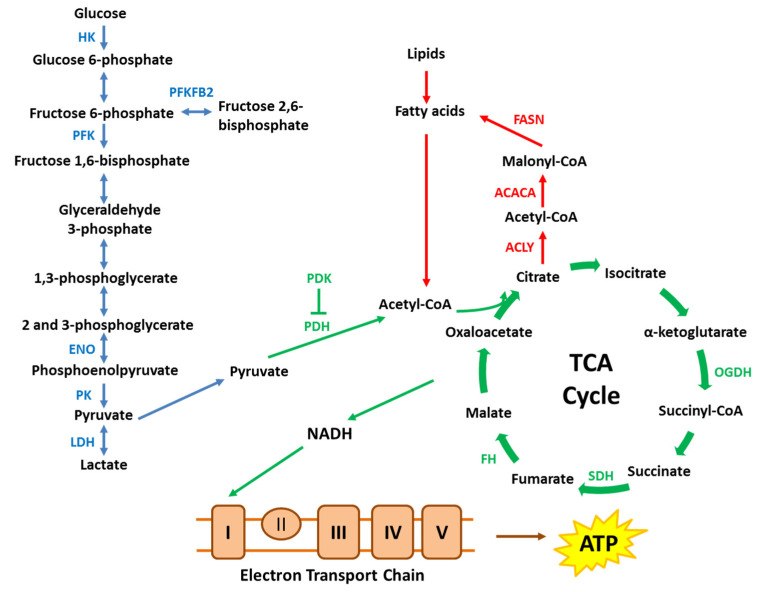
A simplified schematic of key metabolic pathways. Cancer cells alter key enzymes in metabolic pathways, including glycolysis, tricarboxylic acid (TCA) cycle, electron transport chain and lipid metabolism to promote cancer cell survival and proliferation. ACACA, acetyl-CoA carboxylase α; ACLY, ATP citrate lyase; ENO, enolase; FASN, fatty acid synthase; FH, fumarate hydratase; HK, hexokinase; LDH, lactate dehydrogenase; OGDH, oxoglutarate dehydrogenase; PDH, pyruvate dehydrogenase; PDK, pyruvate dehydrogenase kinase; PFK, phosphofructokinase; PFKFB2, 6-phosphofructo-2-kinase/fructose-2,6-bisphosphatase; PK, pyruvate kinase; SDH, succinate dehydrogenase.

Given the critical importance of novel therapeutic approaches for prostate cancer, further understanding in the pathophysiology of prostate cancer is essential. It is well known that androgen mediates prostate growth and development through androgen receptor (AR), which is a principal therapeutic target in androgen-dependent prostate cancer. Several studies have shown that AR signaling alters anabolic and catabolic metabolism in prostate cancer cell lines [7,8,9,10]. In addition, Tat interactive protein 60 kDa (Tip60), an important coactivator of the AR [11], has also been shown to regulate energy metabolism in cells [12,13,14]. In this review, we summarize the AR- and Tip60-mediated metabolic pathways (Figure 1) to show their potential as novel therapeutic targets in androgen-dependent prostate cancer.

## 2. Androgen Receptor Activation through Tip60 Interaction

**Figure 2:**
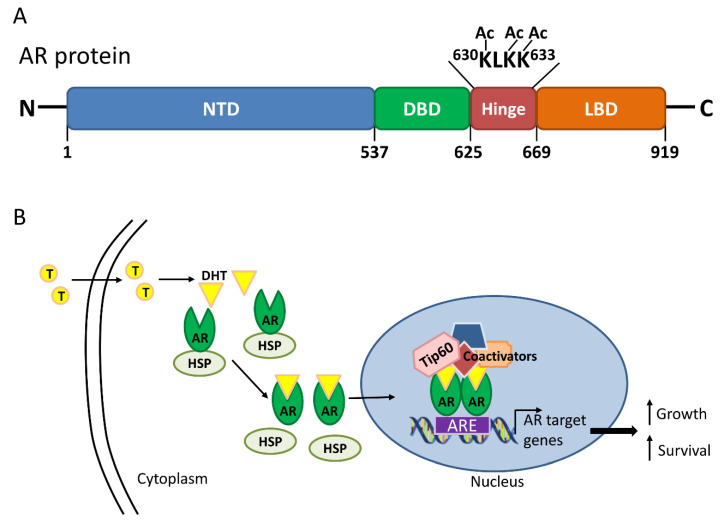
Androgen receptor (AR) structure and activation in the presence of androgen. (**A**) The AR consists of 919 amino acids with four distinct domains, including the N-terminal domain (NTD), DNA-binding domain (DBD), hinge and ligand-binding domain (LBD). Direct acetylation (Ac) by histone acetyltransferases, including Tat interactive protein 60 kDa (Tip60), on the lysine residues of the KLKK motif within the hinge domain is critical for AR activation; (**B**) Upon binding of dihydrotestosterone (DHT), the metabolized form of testosterone (T), the AR dissociates from the heat shock protein (HSP), translocates into the nucleus, dimerizes and binds to the androgen response element (ARE) on AR target genes. Other coactivators, including Tip60, are also recruited for the transcription of AR target genes to sustain cell growth and proliferation.

The AR is an androgen-responsive transcription factor that belongs to the nuclear hormone receptor family. The AR contains four distinct functional domains, including N-terminal, DNA-binding, hinge and ligand-binding domains (LBD) (Figure 2A) [15]. LBD at the C-terminal participates in androgen binding, receptor homodimerization and transcriptional activation [15]. Upon binding of ligands such as testosterone or dihydrotestosterone (DHT), the AR translocates into the nucleus and binds to the androgen response element (ARE) on AR target genes. The recruitment of coactivators by the nuclear AR leads to the transcription of target genes that are critical for the development, growth and survival of prostate cells (Figure 2B) [16].

Tip60, a histone acetyltransferase (HAT) from the MOZ, Ybf2/Sas3, Sas2, and Tip60 (MYST) family, has been shown to interact with the LBD of the AR [11]. Although this interaction is ligand-independent, it can be enhanced in the presence of ligands such as DHT as shown in the yeast two-hybrid system [17]. Within the LBD of the AR, glycine-743 appears to be important for Tip60-AR interaction since its substitution by valine disrupts the integrity of LBD, thereby altering its interaction with Tip60 [17]. In addition, nuclear receptor box within the LXXLL motif of Tip60 is essential for AR interaction since deletion of LXXLL motif failed to induce AR activation after stimulation with mibolerone, a synthetic androgen [17]. Moreover, it has been demonstrated that leucine-497 of Tip60 LXXLL motif is crucial to produce a hydrophobic interface that favors interaction with the LBD of the AR [18]. In fact, Tip60 nuclear receptor box mutant, formed by replacing leucine with alanine, failed to interact with the LBD of the AR and thus, no activation of the AR was observed even in the presence of androgen [17].

Similar to other nuclear receptors, the AR is highly modulated by coactivators and corepressors. HATs, including Tip60, p300 and p300/cAMP-response element-binding protein-associated factor (P/CAF) [19], as well as histone deacetylases (HDACs), such as HDAC1 [20] and Sirtuin 1 [21], regulate the AR through acetylation and deacetylation, respectively [22]. Acetylation is important for AR activation since upregulation of HDAC1 leads to a reduction in AR activity in AR-transfected COS-7 cells [20]. Trichostatin A, a potent HDAC inhibitor, prevents the inhibition of AR activity induced by the upregulation of HDAC1 in these cells [20]. The repression of AR activity by HDAC1 can also be abolished with increasing amounts of transfected Tip60, indicating that Tip60 can compete and overcome the effects of HDAC1 and that acetylation is critical for AR activation [20]. Given that HAT-defective mutant Tip60 is unable to acetylate the AR in COS-7 cells transfected with AR [20], it is likely that Tip60 acetylates the AR directly.

AR is preferentially acetylated on three lysine residues (Lys-630, Lys-632, and Lys-633) within the short KLKK motif of the hinge domain by Tip60 [20], p300 and P/CAF [19]. When the lysine residues were substituted by arginine, which mimics a non-acetylated phenotype of the AR, the mutated AR proteins were mainly localized in the cytoplasm despite androgen stimulus in the androgen-dependent LNCaP prostate cancer cells [23]. However, when lysine was substituted by glutamine within the KLKK motif hinge region of the AR to mimic an acetylated phenotype of the receptor, the AR was mainly located in the nucleus even with androgen starvation [23], suggesting that these lysine residues are critical for AR activation. Another study, which replaced lysine residues with alanine on Lys-630 or both Lys-632 and Lys-633 of the AR [20], showed a dramatic decrease, but not elimination, of Tip60-induced AR activation in the presence of synthetic androgen R1881 in COS-7 cells co-transfected with Tip60 and AR [20]. Together, these findings indicate that acetylation of the three lysine residues by HATs, including Tip60, are important for the full activation of AR activity.

## 3. Roles of the AR in Prostate Cancer Energy Metabolism

**Figure 3:**
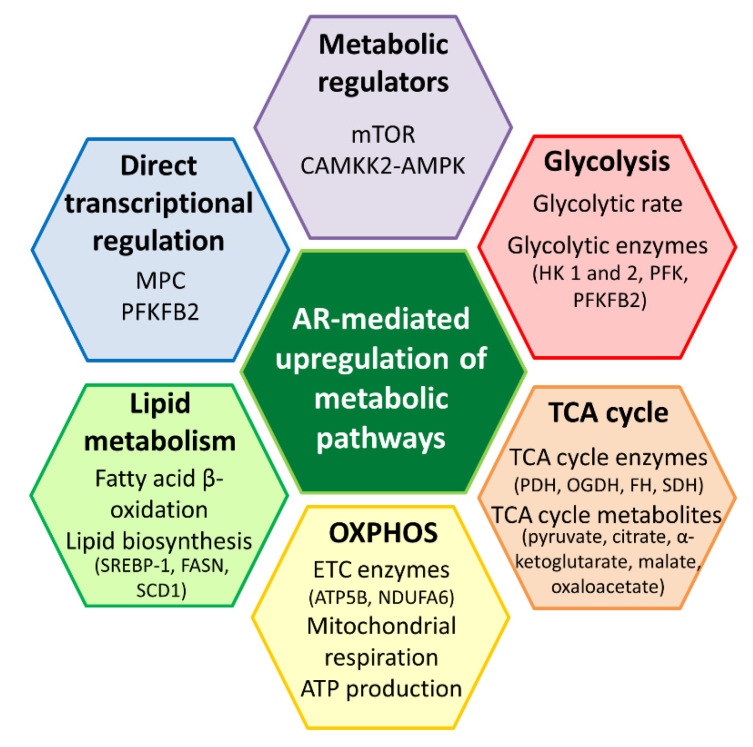
Metabolic roles mediated by the androgen receptor (AR) in prostate cancer. The AR upregulates various metabolic pathways to promote the survival of prostate cancer cells. It is likely that the AR modulates metabolic pathways, such as glycolysis, tricarboxylic acid (TCA) cycle, oxidative phosphorylation (OXPHOS) and lipid metabolism through key metabolic regulators, including mTOR and CAMKK2-AMPK. The AR could also regulate metabolic enzyme and transporter, such as PFKFB2 and MPC through direct transcriptional regulation. AR is an important metabolic regulator in prostate cancer. AMPK, AMP-activated protein kinase; ATP5B, ATP synthase subunit beta; CAMKK2, calcium/calmodulin-dependent kinase kinase 2; FASN, fatty acid synthase; FH, fumarate hydratase; HK, hexokinase; MPC, mitochondrial pyruvate carrier; mTOR, mammalian target of rapamycin; NDUFA6, NADH dehydrogenase (ubiquinone); OGDH, oxoglutarate dehydrogenase; PDH, pyruvate dehydrogenase; PFK, phosphofructokinase; PFKFB2, 6-phosphofructo-2-kinase/fructose-2,6-bisphosphatase; SCD1, stearoyl-CoA desaturase 1; SDH, succinate dehydrogenase; SREBP-1, sterol regulatory element-binding protein-1.

It has been well established that AR activation is essential for cell viability and is associated with an increase in cell proliferation and invasion [24]. These processes are metabolically demanding, and cancer cells are known to alter and adapt their metabolism in order to produce more energy and building blocks such as nucleotides, proteins and lipids to sustain aberrant proliferation [25]. Although the identification of a metabolic phenotype of cancer cells is not recent, the effects of androgen/AR-signaling on prostate cancer cell metabolism are emerging and remain to be elucidated. Several studies that have explored the potential roles of the AR in these cancer cells indicate that the AR regulates glucose metabolism, including glycolysis, TCA cycle and oxidative phosphorylation (OXPHOS), as well as lipid metabolism in androgen-dependent prostate cancer (Figure 3).

### 3.1. Molecular Effects of the AR on Glucose Metabolism

Numerous cancer cell types have been shown to exhibit the Warburg effect or aerobic glycolysis where the glycolytic rate is increased and pyruvate is preferentially converted into lactate, even under aerobic conditions [26]. Since the net energy production through glycolysis is much lower compared to OXPHOS, it was postulated that cancer cells undergo these metabolic alterations due to defective mitochondria [26]. However, some studies have suggested that this metabolic adaption is triggered by the need for biomass production to sustain cell proliferation and since aerobic glycolysis provides more anabolic intermediates, it is more advantageous for cells that are actively proliferating, including stem and cancer cells [27]. In light of this notion, it is highly likely that other metabolic pathways such as the TCA cycle and electron transport chain are functional in cancer cells. Indeed, growing evidence indicates that numerous types of cancer cells do have functional mitochondria and that OXPHOS plays an important role in the survival of cancer cells [28]. Based on these new insights, several studies have explored the roles of the AR in glucose metabolism, including glycolysis, the TCA cycle and OXPHOS using in vitro models of androgen-sensitive prostate cancer [7,9,10,29,30,31].

The AR has been shown to increase glucose uptake in LNCaP cells [9,10], which is in line with clinical observations in patients undergoing cancer screening using ^18^F-fluorodeoxyglucose (a glucose analog that is taken up by metabolically active tumor cells) through positron emission tomography/computed tomography. This could be attributed to the AR-mediated upregulation of glucose transporters (GLUTs) in prostate cancer cells, as demonstrated in several studies, including GLUT1 [9,32], GLUT3 [33] and GLUT12 [34]. GLUTs are known to be mediated by metabolic regulators, such as AMP-activated protein kinase (AMPK) [35,36] and hypoxia-inducible factor 1 alpha (HIF-1α) [37]. Interestingly, GLUT1 shares sequence homologies with the LBD of the AR [38], suggesting that androgen could bind to GLUT1. Indeed, androgen has been shown to bind to the external surface of GLUT1 [38] and thus, it is likely that androgen could modulate glucose uptake through GLUT1 directly.

Consistent with other cancer types, an increase in glucose uptake or transporter is also associated with elevated glycolytic activity and emerging evidence has established the significance of the AR in glycolysis [7,9,10]. This is demonstrated by the increase in extracellular acidification rate, an indicator of glycolytic activity, following androgen treatment in androgen-dependent LNCaP and VCaP cells [7,29,30,31]. The VCaP cell line was established from metastatic bone cancer and has amplified expression of the wild-type AR [39,40]. LNCaP cells, which were derived from a patient lymph node metastasis, have a mutated AR containing the threonine to alanine mutation at amino acid 877 within the LBD; this mutation confers broadened ligand responsiveness and activation by a variety of hydrophobic biomolecules [39,41]. The importance of androgen is further exemplified by the observation that androgen increased the glycolytic activity dose-dependently in both LNCaP and VCaP cells [7]. Additionally, some studies have identified several key glycolytic enzymes that were upregulated by androgens in prostate cancer models, including hexokinase (HK) 1 and 2, phosphofructokinase (PFK), pyruvate kinase (PK) and 6-phosphofructo-2-kinase/fructose-2,6-bisphosphatase (PFKFB2) [9,31]. The activity of HK2 promoter was increased by androgen in LNCaP cells, consistent with an increase in HK2 protein level [10]. Interestingly, the induction of HK2 was attenuated by a protein kinase A (PKA) inhibitor, indicating that androgen-mediated upregulation of HK2 expression is dependent on PKA signaling [10]. In the same study, a chromatin immunoprecipitation (ChIP) assay revealed that androgen increased the recruitment of the AR to the promoter of the PFKFB2 in LNCaP cells, suggesting that a functional ARE is present at the promoter. The relevance of the ARE in the induction of PFKFB2 expression is evident since mutation in the ARE of the PFKFB2 promoter abolished the effects of androgen [10]. Collectively, these studies exemplify the critical influence of the AR in glycolysis.

### 3.2. Molecular Effects of the AR on Mitochondrial Function

Given the vast interest in the roles of mitochondria in tumor initiation and progression, the effects of the AR on the TCA cycle and OXPHOS in androgen-dependent prostate cancer cell lines have also been explored. In addition to glycolysis, AR activation also promotes mitochondrial respiration in LNCaP and VCaP cells [7,29,30], as shown by a dose-dependent increase in oxygen consumption rate in the presence of androgens [7]. This suggests that the increase in glucose uptake and glycolysis induced by the AR also results in an increase in energy metabolism through the TCA cycle and OXPHOS. Indeed, based on steady state metabolomics analysis, androgen treatment increased the levels of several TCA cycle metabolites, including pyruvate, citrate, α-ketoglutarate, malate, and oxaloacetate in LNCaP cells [7,8]. Additional investigations using isotope-labelled glucose also found isotope enrichment in TCA cycle metabolites following androgen treatment [8], further corroborating this notion. Given that the TCA cycle intermediates are also the precursors in several anabolic pathways, an increase in these intermediates does not necessarily result in more ATP production. Therefore, intracellular ATP levels have also been determined and androgen was found to increase ATP levels dose-dependently [7], highlighting the roles of the AR in mediating mitochondrial respiration in androgen-dependent prostate cancer.

The increase in TCA cycle metabolites and intracellular ATP levels could be attributed to AR-mediated transcriptional regulation of metabolic genes in the mitochondria. The AR has been shown to induce the gene expression of key enzymes involved in the TCA cycle, including pyruvate dehydrogenase (PDH) [9,30], α-ketoglutarate dehydrogenase (OGDH) [8], fumarate hydratase (FH) [30] and succinate dehydrogenase (SDH) [30], as well as mitochondrial complexes (such as ATP synthase (ATP5B) [30] and NADH dehydrogenase (ubiquinone) (NDUFA6) [30]). Additionally, a recent study has revealed that mitochondrial pyruvate carrier (MPC), which governs the transport of pyruvate, the end product of glycolysis, across the inner mitochondrial membrane for incorporation into the TCA cycle, is involved in tumor growth in both hormone-responsive and castrate-resistant prostate cancer [42]. Interestingly, the protein levels of MPC, specifically MPC2, were upregulated by androgens in LNCaP cells and the induction was abolished by the anti-androgen, enzalutamide [42]. Two AR binding sites have been identified on the locus of MPC2 and the ChIP assay has established androgen-dependent AR recruitment to both sites, indicating that MPC2 is mediated by the AR through direct transcriptional regulation [42]. Furthermore, androgens have also been shown to upregulate mitochondrial biogenesis by inducing peroxisome proliferator-activated receptor gamma coactivator 1-alpha (PGC-1α) in LNCaP cells [7]. The increase in mitochondrial number is likely to contribute to the increase in mitochondrial-mediated energy metabolism, such as through the TCA cycle and OXPHOS. 

In addition to direct transcriptional regulation of metabolic enzymes, the AR can also interact with other regulators of metabolic pathways. For instance, calcium/calmodulin-dependent kinase kinase 2 (CAMKK2) has been identified as one of the targets of the AR in both androgen-sensitive and castrate-resistant prostate cancer cell lines since the AR was recruited to the promoter of CAMKK2 in both stages of disease [9,43,44]. Further analysis using ChIP has established that CAMKK2 is indeed a direct target of the AR in prostate tumor biopsies from human patients [9]. CAMKK2 has been shown to phosphorylate AMPK [45], an important energy sensor that governs glucose and lipid metabolism. Inhibition of CAMKK2 chemically or using siRNA was associated with reductions in the levels of phosphorylated AMPK, glucose uptake and PFK activity, and consequently, the proliferation of LNCaP cells was greatly decreased [9]. Taken together, these findings are in accord with studies that have consistently identified that CAMKK2 was overexpressed in human prostate tumor biopsies [9,43], highlighting the significance of AR-CAMKK2-AMPK signaling in mediating energy metabolism in prostate cancer. 

Furthermore, mammalian target of rapamycin (mTOR), another key regulator of energy metabolism and cell proliferation, has been shown to be activated by the AR in LNCaP cells [9,29,46]. The activation of mTOR was inhibited by the AR antagonist, bicalutamide [29,46], revealing the importance of the AR in mTOR activation. The upregulation of AR-mediated metabolic genes that are involved in glycolysis and OXPHOS, including HK2, ATP5L and NDUFA6, was abrogated in the presence of mTOR inhibitors [29]. Additionally, glucose uptake, extracellular acidification rate, mitochondrial respiration and mitochondrial content induced by the AR were also significantly abolished by mTOR inhibitors [29], suggesting that mTOR is responsible for at least a subset of AR-driven metabolic reprogramming.

### 3.3. Molecular Effects of AR Activation on Lipid Metabolism

Cell proliferation and invasion are metabolically demanding, and in addition to energy production, building blocks such as lipids, which constitute both the plasma membrane and internal membrane, are also fundamental to sustain the aberrant proliferation that occurs in prostate cancers. Several studies have consistently shown that the AR upregulated the mRNA and protein levels of anabolic enzymes that are involved in *de novo* lipid synthesis, including ATP citrate lyase (ACLY), acetyl-CoA carboxylase α (ACACA), fatty acid synthase (FASN) and fatty acid elongase 7 (ELOV7) in LNCaP cells [8,9,10,29]. Functionally, the upregulation of these lipogenic enzymes contributed to an increase in lipid synthesis, as supported by the incorporation of carbon-14 from ^14^C-glucose into lipids [10] and the observation that the AR increased lipid droplet accumulation in LNCaP cells [8,47].

In addition to direct transcriptional regulation of lipogenic enzymes, the AR can also regulate sterol regulatory element-binding protein-1 (SREBP-1), a key transcription factor in fatty acid and cholesterol biosynthesis. Upon androgen stimulation, the expression of SREBP-1 was increased along with the induction of SREBP-1 target genes that are involved in lipid metabolism, including FASN and stearoyl-CoA desaturase 1 (SCD1) in LNCaP cells [48]. Interestingly, the expression of SREBP-1 and its downstream targets, FASN and SCD1, are strongly correlated to the progression of prostate cancer [48,49]. The inhibition of SREBP-1 directly or through mTOR abolished the induction of FASN and SCD1 as well as androgen-mediated lipid droplet accumulation in LNCaP cells [48], highlighting the contributions of SREBP-1 in androgen-driven lipid biosynthesis.

Apart from the induction of lipid biosynthesis, the AR also promotes the utilization of fatty acids for energy production given that androgens increased the β-oxidation of ^14^C-labelled fatty acids, such as palmitate and oleate, in a dose-dependent manner in LNCaP and VCaP cells [7,8]. More importantly, co-treatment with etomoxir, an inhibitor of carnitine palmitoyltransferase I, which is involved in the rate-limiting step of β-oxidation, inhibited the growth of LNCaP cells [7]. Thus, β-oxidation of fatty acids appears to be fundamental for androgen-mediated prostate cancer cell growth, which concurs with previous studies that fatty acid metabolism plays an important role in prostate cancer [50]. Taken together, the anabolic and catabolic effects of the AR on lipid metabolism could contribute to metabolic plasticity that allows prostate cancer cells to sustain proliferation through lipid biosynthesis to provide building blocks and utilization of alternate substrates, such as fatty acids, for energy production. Overall, these studies have unraveled the novel functions of the AR in mediating glucose and lipid metabolism directly or indirectly in androgen-dependent prostate cancer (Figure 3) and provided a framework to exploit the AR or the associated downstream pathways for therapeutic purposes.

## 4. Roles of Tip60 in Cancer Energy Metabolism

In addition to its classical effect in regulating gene transcription and DNA damage response, several studies have revealed that Tip60 has a critical role in determining cell fate. Although the metabolic roles of Tip60 in prostate cancer remain to be elucidated, several studies have indicated that Tip60 affects cell survival indirectly by modulating key regulators involved in the process of metabolic stress and rewiring, such as c-Myc, HIF-1α and p53, in other cancer types [12,13,51,52]. Furthermore, Tip60 has been shown to modulate the activity of key enzymes in gluconeogenesis and glycolysis directly, specifically phosphoenolpyruvate carboxykinase (PEPCK) and pyruvate kinase, through acetylation [14,53]. 

### 4.1. Molecular Effects of Tip60 on Glucose Metabolism and Mitochondrial Function in Cancer

Major oncogenes, such as c-Myc and HIF-1α, are reported to be master inducers of cancer glycolysis through direct or indirect transactivation of cancer glycolytic genes [54]. In normal cells, c-Myc is induced upon growth factor stimulation, and is involved in many biological processes, including proliferation, cell cycle progression, cell growth, metabolism, angiogenesis, differentiation, cell adhesion and mobility [55]. In cancer cells, c-Myc regulates energy metabolism and ribosomal biogenesis, which are required for rapid proliferation, independent of growth factor stimulation [56].

c-Myc has a key role in glycolysis by inducing the expression of HK2, lactate dehydrogenase A (LDH-A) and pyruvate dehydrogenase kinase 1 (PDK1) [57,58]. HK catalyzes the first step of glycolysis and the three isoforms that have been identified differ in their catalytic and regulatory properties, as well as in their subcellular localization [59]. HK2 is the predominantly expressed isoform in cancer and promotes glycolysis by suppressing the negative feedback provided by glucose-6-phosphate [60,61]. HK2 interacts with the voltage-dependent anion channel on the outer mitochondrial membrane and inhibits mitochondria-induced apoptosis by reducing the release of cytochrome C and Bax [62]. LDH-A plays a key role in regulating glycolysis by catalyzing the conversion of pyruvate to lactate, the final step of anaerobic glycolysis [63]. Therefore, upregulation of LDH-A facilitates the efficiency of anaerobic glycolysis in cancer cells and reduces their dependence on oxygen, which contributes to adaptation upon metabolic stress [63]. PDK1, by inactivating PDH, the enzyme that catalyzes the conversion of pyruvate into acetyl-CoA, stimulates glycolysis and diminishes mitochondrial respiration [57,58]. c-Myc also promotes glycolysis by increasing the expression of GLUT1 and glycolytic enzymes (such as PFK and enolase 1), thereby enhancing glucose uptake and its breakdown [64]. Tip60 has been shown to acetylate c-Myc, which results in a dramatic increase in its protein stability, as shown by the three-fold increase in its half-life in human lung carcinoma cells (H1299) overexpressing Tip60 and c-Myc [12]. Tip60, by increasing the steady-state levels of c-Myc, contributes to the upregulation of genes involved in glycolysis, and thus promotes adaptation to metabolic stress in cancer cells. 

HIF-1α is a transcription factor involved in adaptive responses upon reduced oxygen availability and mediates energy metabolism under hypoxic conditions [65,66]. HIF-1α has been shown to promote glycolysis by upregulating the transcription of several glycolytic enzymes in a greater manner during hypoxia than normoxia [67,68]. In addition, HIF-1α also contributes to the metabolic adaptation of cancer cells by upregulating vascular endothelial growth factor (VEGF) and GLUT1 during hypoxia [69,70]. VEGF is the key mediator of angiogenesis in many cancers, a process that is critical to sustain nutrient and oxygen supply for tumor survival and growth [71]. HIF-1α activation also inhibits OXPHOS by upregulating genes such as PDK1 and LDH-A to reduce the entry of pyruvate into the TCA cycle [72]. The role of Tip60 in HIF-1α activation has been implicated in hypoxia based on the expression of HIF-1α-dependent genes in colorectal cancer cells through mechanisms that are independent of HIF-1α stabilization [13]. Tip60 is essential for histone acetylation and RNA polymerase II activation upon hypoxia at the HIF-1α-dependent gene loci, which favors their transcription [13]. In fact, approximately 25% of HIF-1α-dependent genes rely on Tip60 for full expression during hypoxia, demonstrating that Tip60 has a substantial contribution to HIF-1α-driven expression [13]. Thus, Tip60 appears to be instrumental in the metabolic adaptation that cancer cells acquire to overcome metabolic stress and to survive under low oxygen and nutrient conditions. Specifically, Tip60 could indirectly increase glycolysis, decrease OXPHOS and promote angiogenesis to provide energy and nutrients to sustain tumor development and growth [12,13].

On the other hand, Tip60 may have a protective role against cancer development by inducing apoptosis through the activation of p53 [73,74,75]. p53 is a tumor suppressor that responds to DNA damage or checkpoint failure by either arresting the cells in G1 phase for damage repair or triggering apoptosis to eliminate the damaged cells [76,77]. p53 induces apoptosis mainly through transcriptional induction of the pro-apoptotic Bcl-2 family member, p53 upregulated modulator of apoptosis (PUMA) [78]. The ability of Tip60 to activate p53 by acetylation at K120, which promotes the expression of PUMA, has been reported in human colon cancer cells upon DNA damage [51]. Under metabolic stress induced by glucose deprivation, K120 in p53 is also critical for its activation/acetylation induced by Tip60 in hepatocellular cancer cells [52]. K104 in Tip60 is indispensable for Tip60 activation and the induction of p53-mediated PUMA expression [52]. In fact, mutation at this lysine acetylation site hindered the binding of Tip60 to the human NuA4 complex, suppressed the acetyltransferase activity of Tip60 and inhibited the expression of pro-apoptotic genes upon glucose starvation [52]. These studies suggest a protective effect of Tip60 in cancer, and its downregulation in some tumors [79,80] may explain a mechanism by which cancer cells adapt to metabolic stress.

**Figure 4:**
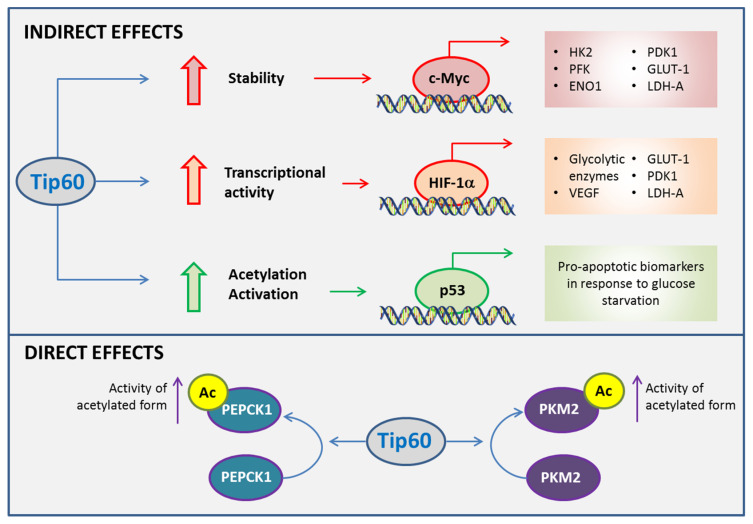
Roles of Tip60 in cancer metabolism. Tip60 influences cell fate in metabolic adaptations of cancer cells indirectly by impacting on transcriptional activities of c-Myc, hypoxia inducible factor 1α (HIF-1α) and p53. Tip60 contributes to the metabolic plasticity of cancer cells upon metabolic stress by inducing the activities of glycolytic and gluconeogenic enzymes directly. ENO1, enolase 1; GLUT1, glucose transporter 1; HK2, hexokinase 2; PDK1, pyruvate dehydrogenase kinase 1; LDH-A, lactate dehydrogenase A; PEPCK1, phosphoenolpyruvate carboxykinase 1; PFK, phosphofructokinase; PKM2, pyruvate kinase isoform M2; VEGF, vascular endothelial growth factor.

Collectively, the data suggests that Tip60 has an indirect role in the adaptation of cancer cells to metabolic stress by modulating the transcriptional activities or levels of c-Myc, HIF-1α and p53 (Figure 4). These factors result in contrasting cell fate given that c-Myc and HIF-1α are activators of the pro-survival pathways whereas p53 triggers the pro-apoptotic cascade. Thus, Tip60 may exert a dual effect in cancer progression depending on the molecular pathway that it activates. However, more studies are still needed to further understand the molecular cues and/or tumor micro-environment that influences the effects of Tip60 upon metabolic stress.

Tip60 has been shown to regulate glycolysis and gluconeogenesis through direct modulation of enzymatic activities. The gluconeogenesis pathway is usually inhibited in cancer because it antagonizes glycolysis, a metabolic pathway that is highly active in cancer cells to maintain sufficient glycolytic intermediates and NADPH for cell proliferation [81]. Both isoforms of PEPCK, cytosolic PEPCK1 and mitochondrial PEPCK2, catalyze the conversion of oxaloacetate to phosphoenolpyruvate, which is the initial step in gluconeogenesis [82]. In addition to gluconeogenesis, PEPCK also acts as a cataplerotic enzyme to support cancer cells under nutrient depletion by utilizing non-carbohydrate precursors for anabolic biosynthesis [83,84]. It has been shown that knocking down Tip60 had no effect on the endogenous PEPCK1 level in HepG2 cells, but it dramatically diminished the acetylation fraction of PEPCK1 [14]. In addition, the amount of glucose secreted into glucose-free medium significantly declined in Tip60 knockdown HepG2 cells, indicating that Tip60 modulates the enzymatic activity of PEPCK1 directly through acetylation [14]. Therefore, it is likely that Tip60 could exert a pro-carcinogenic effect by promoting metabolic plasticity for tumor survival and growth via activating PEPCK. This effect of Tip60 would not be restricted to gluconeogenic organs, such as liver and kidney, since PEPCK is distributed ubiquitously and highly overexpressed in cancers [84]. 

PK is the enzyme involved in catalyzing the conversion of phosphoenolpyruvate and ADP to pyruvate and ATP, the last step of glycolysis [85]. The M2 isoform of PK (PKM2) supports anabolic metabolism and is expressed in both cancer and normal cells [85]. PKM2 has a key role in cancer progression either through its effect on glycolysis or through its non-canonical functions [85,86,87]. PKM2 has been shown to promote glycolysis by inactivating PDH, a rate-limiting step that governs the entry of pyruvate into the TCA cycle [85,86]. Non-canonical PKM2 functions involve the regulation of gene expression and cell cycle progression via protein–protein interactions and protein kinase activity [86,87]. Tip60 has been shown to acetylate PKM2 in HeLa cells co-transfected with Tip60 and PKM2 [53]. This post-translation modification increases PKM2 activity, consistent with that observed previously with the histone acetyltransferase p300 in recombinant PKM2 [88]. The direct effects of Tip60 on the activity of key glycolytic and gluconeogenic enzymes that contributes to the metabolic adaptation of cancer cells are summarized in Figure 4.

### 4.2. Tip60 as Potential Therapeutic Target in Prostate Cancer

Tip60 has been shown to be instrumental for the activation of the AR in androgen-sensitive cells [23] and it is highly likely that Tip60 itself also plays an important role in androgen-insensitive cells, since there is a shift in the cellular distribution and localization of Tip60 from predominantly cytoplasmic to nuclear as the disease progresses towards hormone therapy resistance. In fact, in human biopsies of hormone refractory prostate cancer, an increase in Tip60 expression and nuclear accumulation was observed compared to benign prostate hyperplasia and primary prostate cancer, in which a more cytoplasmic distribution was detected [89]. Interestingly, although AR activation mediated by Tip60 is associated with the presence of androgen stimulus, AR activation could be induced even in the absence of androgens when Tip60 is highly overexpressed [23,31]. A recent study has further demonstrated that overexpression of Tip60 alone was sufficient to increase cell proliferation and glycolytic activity, most probably through the stabilization of HIF-1α in LNCaP cells [31]. This suggests that an overexpression of Tip60 with increased nuclear localization contributes to AR activation in an androgen-independent manner, thus promoting the transition of an androgen-responsive stage to castrate-resistant prostate cancer.

Given the critical roles of Tip60 in AR activation, and in various metabolic pathways, targeting Tip60 appears to be a promising therapeutic option for different stages of prostate cancer, including the androgen-independent stage. For instance, cell proliferation induced by androgen was reduced in LNCaP cells when Tip60 was downregulated [23,90] and the silencing of Tip60 has also been shown to decrease the proliferation of CxR cells, an androgen-insensitive LNCaP derivative cell line [23]. Moreover, NU9056 (1, 2-bis (isothiazol-5-yl) disulfane), identified as potent HAT inhibitor with high selectivity against Tip60, has been shown to inhibit cellular proliferation and induce apoptosis in androgen-dependent and independent cell lines. These effects of NU9056 were also accompanied by a decrease in AR and PSA levels [90]. Collectively, these findings indicate that strategies aiming to inhibit or downregulate Tip60 may be beneficial for the treatment of prostate cancer.

Based on the results from in vitro and pre-clinical models, HATs have been increasingly recognized as promising targets for cancer therapeutics. In fact, a clinical trial evaluating the effect of an anti-cancer drug in the progression of Lymphoma in patients with mutations in HATs genes, such as CREBBP and EP300, is being developed at the Memorial Sloan Kettering Cancer Center in the US (NCT02282358). In addition, there are several other clinical trials for epigenetic drugs including prostate cancer patients [91]. Although no direct interventions in human models targeting Tip60 have been performed to date, the relevance of developing clinical trials targeting this HAT relies on the fact that Tip60 has been found to be upregulated in human tumor samples and associated with cancer progression and malignancy. For example, Tip60 was found to be upregulated in malignant pleural mesothelioma compared to benign pleura [92]. Similarly, Tip60 was found to be overexpressed in tumor biopsies of radioresistant prostate cancer compared to the radiosentitive tumors [93]. 

## 5. Conclusions

Studies to date have demonstrated that the AR exerts a regulatory effect on key metabolic pathways beyond its classical role as a hormone receptor during prostate cancer development and progression. The significance of the AR in prostate cancer is evident from the involvement of the AR in a range of metabolic processes, both anabolic and catabolic pathways, to promote the survival of prostate cancer cells. Additionally, Tip60, an important coactivator of the AR, also exhibits instrumental metabolic roles in cancer cells through direct and indirect regulation. Although some preliminary studies have revealed the promising potential of Tip60 as a therapeutic target in androgen-dependent prostate cancer, further studies are warranted to explore the effects of Tip60 inhibition on metabolic pathways that are mediated by Tip60 and the AR in different stages of prostate cancer. Nonetheless, both the AR and Tip60 are undeniably master regulators of the metabolic network in prostate cancer and they continue to constitute potential avenues for novel therapeutic options in androgen-sensitive prostate cancer.
